# Cell-type-specific 3D-genome organization and transcription regulation in the brain

**DOI:** 10.1101/2023.12.04.570024

**Published:** 2023-12-05

**Authors:** Shiwei Liu, Pu Zheng, Cosmos Yuqi Wang, Bojing Blair Jia, Nathan R. Zemke, Bing Ren, Xiaowei Zhuang

**Affiliations:** 1 Howard Hughes Medical Institute, Department of Chemistry and Chemical Biology, Department of Physics, Harvard University, Cambridge, MA, USA; 2 Department of Molecular and Cellular Biology, Harvard University, Cambridge, MA, USA; 3 Bioinformatics and Systems Biology Graduate Program, Medical Scientist Training Program, University of California San Diego, La Jolla, CA, USA; 4 Department of Cellular and Molecular Medicine and Center for Epigenomics, University of California, San Diego School of Medicine, La Jolla, CA, USA

## Abstract

3D organization of the genome plays a critical role in regulating gene expression. However, it remains unclear how chromatin organization differs among different cell types in the brain. Here we used genome-scale DNA and RNA imaging to investigate 3D-genome organization in transcriptionally distinct cell types in the primary motor cortex of the mouse brain. We uncovered a wide spectrum of differences in the nuclear architecture and 3D-genome organization among different cell types, ranging from the physical size of the cell nucleus to the active-inactive chromatin compartmentalization and radial positioning of chromatin loci within the nucleus. These cell-type-dependent variations in nuclear architecture and chromatin organization exhibited strong correlation with both total transcriptional activity of the cell and transcriptional regulation of cell-type-specific marker genes. Moreover, we found that the methylated-DNA-binding protein MeCP2 regulates transcription in a divergent manner, depending on the nuclear radial positions of chromatin loci, through modulating active-inactive chromatin compartmentalization.

The eukaryotic nucleus is the central hub for essential genomic functions, ranging from transcription and gene-expression regulation to the replication of DNA. The morphology and molecular architecture of the cell nucleus are among the most distinct indicators of cell differentiation, aging, and disease progression (1, 2). Decades of imaging and biochemical studies have provided rich insights into the nuclear architecture and chromatin organization across scales (3–9). Recent development of high-throughput sequencing- and imaging-based assays for 3D-genome mapping has substantially advanced our understanding of the chromatin structure in the interphase nucleus with a genome-wide view (3–5, 8–15). Studies using these methods have revealed prominent chromatin structures such as chromatin compartments (16), topologically associating domains (TADs) (17–19), and chromatin loops (20). For example, both sequencing- and imaging-based methods have shown that chromatin in the interphase nucleus are segregated into compartments enriched for active and inactive chromatin marks (termed A and B compartments), with compartment-A and compartment-B chromatin preferentially positioned in the interior and periphery of the nucleus, respectively (16, 21–24).

Recent studies comparing embryonic stem cells and a few differentiated cell types in vitro, as well as comparisons between major cell types in the brain, have revealed changes in A/B compartment arrangements from cell type to cell type, which are correlated with gene expression changes (25–31). However, these effects tend to be subtle and exactly how the A/B compartmentalization affects the transcription activity of chromatin remains unclear. In addition, TADs and loop domains have been observed in different cell types, and the boundaries of these domains vary between cell types (25–27, 29, 31–33). However, genetic manipulations that abolish TADs only have a small effect on transcription activities (34–36). Therefore, what properties of the 3D chromatin organization are related to transcription and what molecular mechanisms underlie these connections remain incompletely understood. Moreover, most of the cell-type-dependence studies of 3D-genome organization have investigated only a small number of cells or cell types. Hence what differences in 3D chromatin organization are present between different cell types in complex tissues and how 3D chromatin organization influences or is influenced by transcription across different cell types remain poorly understood.

3D organization of the genome is regulated by a variety of protein factors that establish, maintain, or modulate chromatin structures (37). For example, cohesin and CTCF are known to regulate chromatin organization, in particular chromatin domains and loops, and the underlying mechanisms have been extensively characterized (38). However, for most transcription regulators, the mechanisms underlying how they control or modulate chromatin organization are less clear. Many of these factors, such as Med1, Brd4, and MeCP2, bind to gene regulatory elements, including histone marks and methylated DNA, and are suggested to drive 3D-genome organization through phase separation (6, 8). Among these proteins, MeCP2, the causal gene of the Rett syndrome – a neurological disorder (39), binds to methylated DNA (40), forms liquid-liquid phase separation (41, 42), and regulates gene transcription (43, 44). Although *Mecp2* deletion or overexpression has been reported to change the size of the nucleus and gross nuclear architecture, as reflected by DAPI and histone mark stains (41, 42, 45, 46), how MeCP2 affects higher-order chromatin structures and how the chromatin-organizing function of MeCP2 regulates transcription mechanistically remain unclear.

In this work, we studied the 3D-genome organization and the effect of MeCP2 on chromatin organization across different cell types in the brain using multiplexed error-robust fluorescence in situ hybridization (MERFISH), a genome-scale imaging method (47). We have previously demonstrated *in situ* cell-type identification in the brain using RNA-MERFISH (48–50) and the determination of 3D-genome organization in cultured cells using DNA-MERFISH (24). Here, we extended DNA-MERFISH to intact tissue samples and combined it with RNA-MERFISH to probe cell-type-specific 3D-genome organization in ~20 major cell types in the primary motor cortex (MOp) of the mouse brain.

Using this approach, we observed multiple levels of differences in the nuclear architecture and chromatin organization between different cell types that were related to transcriptional regulation. First, we showed that the nuclear sizes and chromosome territory sizes varied drastically across different cell types, both of which were strongly correlated with the total transcriptional activity of the cells. Second, we observed cell-type-dependent changes in higher-order chromatin structures. In particular, chromatin exhibited increasingly more pronounced A/B compartments and less pronounced megadomain structures as the overall transcriptional activity increased from non-neuronal cells to inhibitory neurons and then to excitatory neurons. Third, transcriptional activity of differentially expressed genes, as well as the activity of cell-type-specific super-enhancer activities, were correlated with the local enrichment of compartment-A chromatin loci at these genes or super-enhancers across different cell types. Fourth, we observed different nuclear radial positioning of genomic loci in different cell types. In non-neuronal cells, transcriptional activity of genomic loci was strongly correlated with their radial positions in the nucleus, with active and inactive chromatin enriched in the nuclear interior and periphery, respectively. In contrast, active and inactive chromatin in neurons adopted a more intermixed radial positioning in the nucleus. Finally, we found that MeCP2 regulated nuclear radial positioning, local A/B compartmentalization, and transcriptional activity of chromatin loci in a radial-position-dependent and cell-type-specific manner. Notably, upon *Mecp2* deletion, genes located in the nuclear periphery showed increased transcriptional activity whereas genes located near nuclear centers showed decreased transcriptional activity, and these divergent changes can be explained by a common mechanism – loss of MeCP2 weakened A/B-compartment chromatin segregation. Overall, our data provided rich insights into how 3D chromatin organization is connected to transcriptional activities in a cell-type-specific manner in the brain.

## Integrated RNA- and DNA-MERFISH for cell-type-specific chromatin organization mapping

We chose the primary motor cortex (MOp) as a model system to study the cell-type-specific chromatin organization and its relationship to transcriptional regulation. A comprehensive cell-type atlas of the MOp has been generated by the BRAIN Initiative Cell Census Network (51). Specifically, single-cell/nucleus RNA sequencing (sc/snRNA-seq) and epigenomic sequencing studies have extensively characterized cell-type taxonomy as well as the transcriptional regulatory landscape across all identified cell types in the MOp, including the transcriptomic, chromatin accessibility, and DNA-methylation profiles of each cell type (52–54). In parallel, we have generated a spatially resolved cell atlas of the MOp by RNA-MERFISH, reporting a comprehensive list of cell types and their spatial organization in this brain region (50). Here, we sought to develop a platform that combines RNA-MERFISH for resolving transcriptionally defined cell types and DNA-MERFISH for characterizing chromatin structures in single cells to investigate the relationship between cell-type-specific chromatin organization and gene-expression regulation.

We introduced several modifications to our previous MERFISH protocols to facilitate the combination of RNA- and DNA-MERFISH. To preserve 3D chromatin structures in their native environment, we omitted the gel-embedding-based tissue clearing procedures (Materials and Methods), which were previously used in RNA-MERFISH characterizations of MOp cell types (50). To compensate for the decrease in signal-to-noise ratio resulted from omitting tissue clearing, we used an adaptor-probe approach to boost signal, which utilizes an adaptor probe to link dye-labeled readout probes to encoding probes, the latter in turn are bound to target RNA or DNA (24). This approach allows more dye-labeled readout probes to be linked to each encoding probe and hence increases signals from individual mRNA molecules and genomic loci. Additionally, we employed photobleaching to reduce autofluorescence of the tissue slices (49). With these modifications, we performed RNA-MERFISH measurements on coronal mouse brain sections encompassing the MOp (fig. S1). Subsequently, we conducted DNA-MERFISH on the same tissue sections and registered the DNA and RNA images to obtain transcriptional profiles and chromatin 3D structures in the same cells (fig. S1).

To systematically study the cell-type-dependent chromatin structures, we designed encoding probe libraries to target cellular RNAs and chromatin in the following manner. For RNA-MERFISH, we utilized the same MOp encoding-probe library as in our previous work, targeting 242 marker genes for cell-type identification (50). For DNA-MERFISH, we designed three different encoding-probe libraries respectively targeting three groups of genomic loci (1981 loci in total): (1) 988 loci evenly distributed across the mouse genome with ~2.5Mb spacing; (2) 28 loci centered around transcription start sites (TSSs) for representative marker genes for the major cell types in the MOp; (3) 965 loci centered around candidate super-enhancers, genomic regions that comprise multiple putative enhancers and have been implicated in cell-type and gene-expression specification (55, 56). We selected cell-type specific super-enhancers using the published single-nucleus Assay for Transposase-Accessible Chromatin using Sequencing (snATAC-seq) dataset (52) (Materials and Methods).

**Fig. 1.**
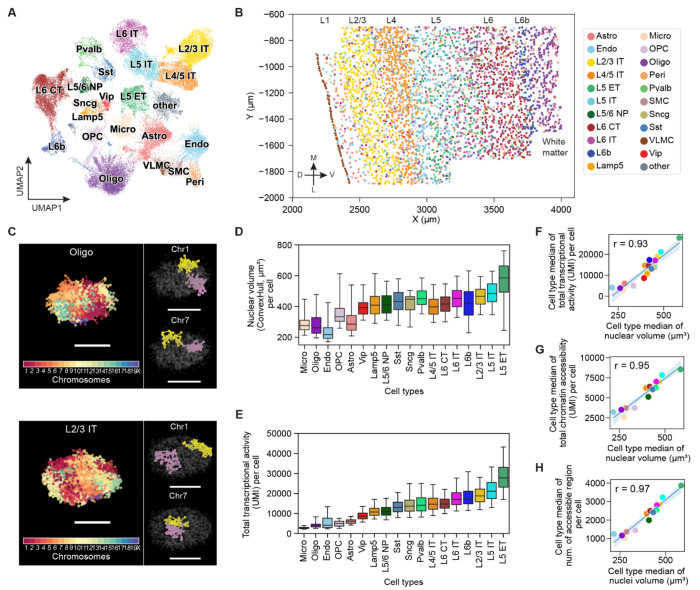
Integrated 3D-genome and transcriptome imaging revealed cell-type-dependent variations of and correlation between cell-nucleus size and transcriptional activity. (**A**) Uniform manifold approximation and projection (UMAP) representation of transcriptionally distinct cell types in the MOp determined by RNA-MERFISH. Cells are colored by their cell-type identities, and the same color code is used in all panels in this figure. Micro: microglia; Oligo: oligodendrocytes; OPC: Oligodendrocyte progenitor cells; Astro: astrocytes; Endo: endothelial cells; Peri: Pericytes; Vip: vip-positive inhibitory neurons; Lamp5: *lamp5*-positive inhibitory neurons; Sst: sst-positive inhibitory neurons; Sncg: sncg-positive inhibitory neurons; Pvalb: pvalb-positive inhibitory neurons; L2/3 IT, L4/5 IT, L5 IT, and L6 IT: L2/3 IT, L4/5 IT, L5 IT and L6 IT excitatory neurons; L5 ET: L5 ET excitatory neurons; L5/6 NP: L5/6 NP excitatory neurons; L6 CT: L6 CT excitatory neurons; L6b: L6b excitatory neurons. (**B**) Spatial map of transcriptionally distinct cell types in a coronal slice of the MOp. Different cortical layers are indicated on top (L1-L6b). D, dorsal; L, lateral; M, medial; V, ventral. (**C**) 3D renderings of chromatin organization in an oligodendrocyte (top) and L2/3 IT neuron (bottom) determined by DNA-MERFISH. Left, all chromosome loci colored according to their chromosome identities; Right, Chr1 and Chr7, whose two homologs are distinctively colored in yellow and purple. Scale bar: 5 μm. (**D**) Boxplot for the distribution of nuclear volume across individual cells in each cell type. Nuclear volume per cell was calculated by the convex hull volume of all chromosome loci. (**E**) Boxplot for the distribution of total transcriptional activity across individual cells in each cell type. Total transcriptional activity per cell was calculated using the sum of unique molecular identifiers (UMIs) from snRNA-seq data of the MOp (52). Cell types are ordered by their rank in the cell-type median of total transcriptional activity per cell. (**F**) Scatterplot of the cell-type medians of total transcriptional activity versus nuclear volume per cell. (**G**) Scatterplot of the cell-type medians of total chromatin accessibility versus nuclear volume per cell. Total chromatin accessibility per cell was calculated using the sum of UMIs per cell from snATAC-seq data of the MOp (52). (**H**) Scatterplot of the cell-type medians of the number of accessible chromatin regions versus nuclear volume per cell. The number of accessible chromatin regions per cell was obtained from snATAC-seq data of the MOp (52). Each dot in (F to G) corresponds to a cell type.

Using this design, we profiled transcription of the 242 genes in 46,340 cells and identified 21 cell types at the subclass level in the MOp and adjacent areas by *de novo* cell clustering (Fig. 1A). These include eight subclasses of excitatory neurons (layer 2/3 (L2/3) intratelencephalic (IT), L4/5 IT, L5 IT, and L6 IT, L5 extratelencephalic (ET), L5/6 near-projection (NP), L6 cortical-thalamic projection (CT), and L6b neurons), five subclasses of inhibitory neurons annotated by their canonical marker genes (*Pvalb*, *Sst*, *Lamp5*, *Vip* and *Sncg*), and eight subclasses of non-neuronal cells (astrocytes, oligodendrocytes, oligodendrocyte progenitor cells (OPC), microglia, endothelial cells, pericytes, vascular leptomeningeal cells (VLMCs), and smooth muscle cells (SMCs)) (Fig. 1, A and B). The overall expression profiles of MOp cells obtained using our modified RNA-MERFISH protocol agreed well with both our previous results from tissue-cleared RNA-MERFISH (50) (fig. S2A) and bulk RNA-seq data (fig. S2B). Our data also showed high reproducibility between experimental replicates (fig. S2C). Hence, the cell types identified using this modified RNA-MERFISH protocol showed a one-to-one correspondence with the cell types determined from our previously published RNA-MERFISH dataset (50), except for the low abundance perivascular macrophages (PVMs) which was not identified here presumably due to fewer cells measured here (fig. S2, D and E).

Next, we determined the 3D-genome organization for each identified cell type. We used 99-bit and 95-bit error-correcting Hamming codes to encode the 988 genome-wide loci and 965 super-enhancer loci, respectively, and imaged these 1953 loci using two back-to-back DNA-MERFISH measurements. In addition, we imaged the 28 marker-gene TSS loci using sequential multi-color DNA-FISH after DNA-MERFISH measurements, all on the same samples. We analyzed the DNA-MERFISH images by adapting the recently reported spatial genome aligner algorithm (57), which allowed us to determine the copy number of chromosomes and trace chromatin loci within each chromosome (Materials and Methods). This approach provided separate 3D structures of homologous chromosomes in individual cells (Fig. 1C). We found that the median pairwise distance between our imaged loci showed a high correlation with the pairwise contact frequency recently measured by single-nucleus methyl-3C (snm3C) (29) (fig. S3), demonstrating that the 3D chromosome structures were well preserved in their native tissue contexts in our integrated RNA- and DNA-MERFISH measurements.

## Cell-type-dependent variations in nucleus and chromosomal territory sizes and their relationship with transcriptional activity

We systematically characterized the size of the cell nucleus in each cell type in the MOp and observed substantially different nuclear sizes for different cell types, with non-neuronal cells, inhibitory neurons, and excitatory neurons exhibiting increasingly larger nuclear sizes (Fig. 1D and fig. S4). Within these major cell classes, different subclasses of cells also showed different nuclear sizes. For example, among excitatory neurons, L5 ET and L4/5 IT showed the largest and smallest nuclear sizes, respectively (Fig. 1D). Even among IT neurons, cells in different cortical layers showed different nuclear sizes (Fig. 1D). These results substantially expanded previous knowledge of different nuclear sizes for different cell types in the cortex (58).

Next, we investigated the relationship between the nuclear size and transcription by using a published snRNA-seq dataset (52) to obtain genome-wide RNA expression levels in different cell types in the MOp. Notably, we observed a strong correlation between the nuclear size and total transcriptional activity of the cell across different cell types (Fig. 1, D to F). Remarkably, the cell type with the largest nuclear sizes, L5 ET, showed a ~10-fold higher total transcript counts than that observed in the cell types with the smallest nuclear sizes, i.e., microglia, oligodendrocytes, and endothelial cells (Fig. 1, D and E). Even among neurons, L5 ET showed a ~3-fold greater total transcript counts than that observed in the neuronal cell types with the smallest nuclear size, the *Vip*-positive inhibitory neurons (Fig. 1, D and E). A similar trend in transcriptional activity changes across cell types was also observed in the human cortex (fig. S5) (49, 59). We also observed a strong correlation between the nuclear size and chromatin accessibility across different cell types (Fig. 1, G and H), using a published snATAC-seq dataset (52) to assess chromatin accessibility.

Next, we investigated whether transcriptional activity was also correlated with the physical sizes of chromosomal territories (fig. S6A). We observed that cell types with larger nuclear sizes generally had larger chromosomal territory sizes, except that most inhibitory neurons exhibited disproportionately larger chromosomal territory sizes compared to excitatory neurons with the same nucleus size (fig. S6, A and B). Consistent with this observation, inhibitory neurons displayed more intermixing of chromosome territories than excitatory neurons (fig. S6C). We also observed a positive correlation between the overall transcriptional activity of the chromosomes and the chromosomal territory sizes across different cell types (fig. S6D), although this correlation was weaker compared to the correlation with the nuclear sizes (Fig. 1F). A possible explanation for this weaker correlation is the stronger influence that each chromosome received from other chromosomes invading its territory in inhibitory neurons, which in turn contributes to transcription regulation. Consistent with this notion, excitatory neurons, which exhibited stronger segregation between chromosomal territories (fig. S6C), also showed higher correlation between transcriptional activity and chromosomal territory sizes (fig. S6E).

## Cell-type-dependent variations in higher-order chromosome structures and their relationship with transcriptional activity

**Fig. 2.**
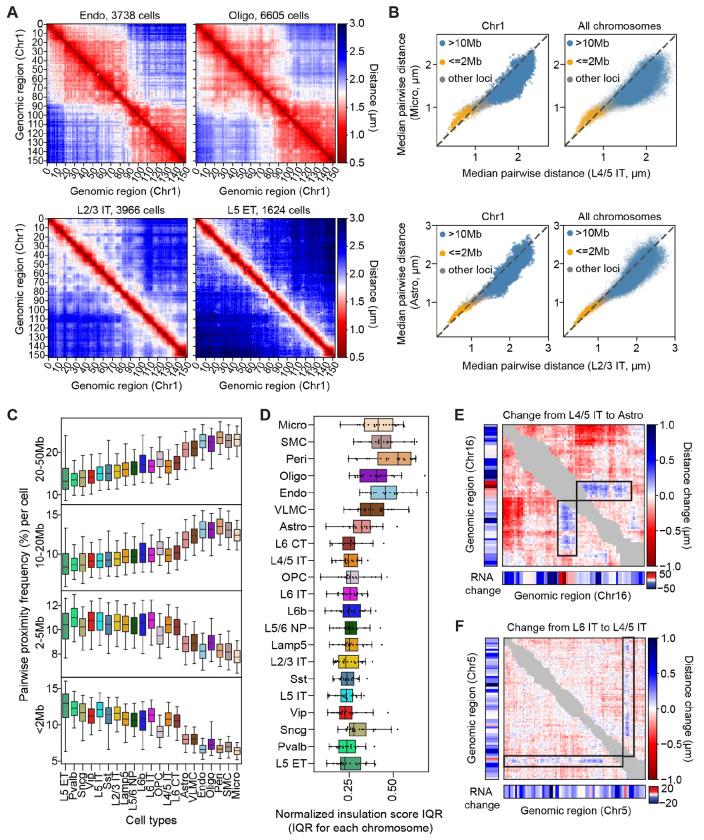
Cell-type-dependent variations in higher-order chromosome structures and transcriptional activity. (**A**) Median pairwise distance matrices for Chr1 (3.7Mb – 98.8Mb) in four example cell types. (**B**) Scatterplots of the cell-type medians of pairwise distances for cis-chromosomal locus pairs in one cell type versus another. Left: all locus pairs from Chr1. Right: all cis-chromosomal locus pairs from all chromosomes. Top: comparison between microglia and L4/5 IT neurons. Bottom: comparison between astrocytes and L2/3 IT neurons. Each dot in the scatterplot is colored according to the genomic distance of the indicated locus pair (orange: <2Mb; blue: >10Mb; light gray: all others). Dashed line indicates equality (y = x). (**C**) Boxplots showing the distributions of cis-chromosomal pairwise proximity frequency between genomic loci across all cells for each cell type, grouped by the genomic distances between individual locus pairs. Pairwise proximity frequency in each cell for the given genomic-distance group was calculated as the ratio of the number of proximal locus pairs (distance < 750nm) per cell over the number of all measured pairs per cell within the genomic-distance group. The center line, box, and whisker represent the median, 25th-75th percentile, and 5th-95th percentile, respectively. (**D**) Boxplots showing the distributions of normalized insulation score interquartile range (IQR) across all chromosomes for each cell type. Normalized insulation scores and the IQR were calculated as described in fig. S8B. (**E**) Comparison of higher-order chromosome organization and transcription in Chr16 (3.7Mb – 97.7Mb) between L4/5 IT and astrocytes. The matrix represents the change in median cis-chromosomal pairwise distance from L4/5 IT to astrocytes. Next to the matrix (left and below) are changes in transcript counts of the corresponding genomic loci from L4/5 IT to astrocytes (derived from snRNA-seq data (52)). Black boxes indicate genomic regions showing local changes between cell types that had an opposite trend to the overall changes of the whole chromosome. Gray elements near the diagonal represent locus-pairs whose genomic distances are <2Mb. (**F**) Similar to (E), but for comparison of higher-order chromosome organization and transcription in Chr5 (3.7Mb – 151.3Mb) between L6 IT and L4/5 IT neurons.

The DNA-MERFISH data allowed us to further determine the higher-order chromosomal organization beyond the sizes of the cell nucleus and chromosomal territories. To this end, we computed the median pairwise distances between imaged loci within individual chromosomes (e.g., Chr1) in different cell types (Fig. 2A). We observed overall increased pairwise cis-chromosomal distances in cells with larger chromosomal territory sizes across different cell types (Fig. 2A; fig. S7). However, closer inspection of our data revealed a deviation from a simple decompaction model (Fig. 2B). Specifically, we observed that locus pairs with genomic distances greater than 10Mb showed increased pairwise spatial distances in cell types with larger chromosomal territory sizes (Fig. 2B), whereas locus pairs with genomic distances smaller than 2Mb often showed the opposite trend (Fig. 2B). We further computed the frequencies of spatial proximity (defined with a 3D distance threshold of 750 nm) between chromatin loci separated by different genomic distances, and observed divergent trends for proximity frequencies between locus pairs with large versus small genomic distances: while genomic loci separated by genomic distances greater than 10Mb showed increased proximity frequencies in cell types with smaller chromosomal territory sizes, the opposite trend was observed for genomic loci separated by genomic distances smaller than 5Mb (Fig. 2C).

In addition to these differences in cis-chromosomal pairwise distances, we also observed large-scale structural differences between different cell types. In particular, when comparing non-neuronal cells and neurons, we observed that chromosomes in non-neuronal cells had a higher tendency to form large-size domains (Fig. 2A and fig. S8A) that resemble the “megadomains” in the transcriptionally inactive X chromosomes (20, 22, 60–62). We quantified the prevalence of these megadomain-like structures using the insulation score calculation (Materials and Methods), and observed more prominent insulation-score peaks, corresponding to the boundaries of megadomain structures, in chromosomes inside non-neuronal cells as compared to neurons (fig. S8B and Fig. 2D). Because non-neuronal cells generally have lower transcriptional activity than neurons (Fig. 1, E and F), we speculate that inactive chromatin may promote the formation of megadomain structures in non-neuronal cells, in a manner similar to how it occurs in inactive X chromosomes (61, 62).

To further explore the relationship between transcription and chromosomal organization, we also examined the differences in median pairwise distance matrices between cell types of different overall transcriptional activities. In most cases, these differential matrices showed unidirectional changes that were consistent with overall differences in chromosomal territory sizes and transcriptional activity: chromosomes in cell types with lower overall transcriptional activity showed more compaction (fig. S9). However, in some cases, we observed exceptions to this general trend (Fig. 2, E and F). For example, when comparing the median pairwise distance matrices of Chr16 between L4/5 IT neurons and astrocytes, we observed clear stripes running against the overall trend of the chromosome (Fig. 2E, indicated by black boxes). The chromatin region associated with these stripes showed notably higher transcriptional activity in astrocytes as compared to L4/5 IT neurons, despite that the chromosome-wise overall transcription activity level was lower in astrocytes (Fig. 2E). The physical distances between this region and the down-stream regions in the chromosome were also larger in astrocytes as compared to those observed in L4/5 IT neurons, even though astrocytes showed a general compaction of this chromosome as compared to L4/5 IT. We observed such “looping-out” of chromatin regions with relatively high transcriptional activity not only when comparing non-neuronal cell types with neurons, but also when comparing different neuronal cell types, for example, in Chr 5 between L4/5 IT and L6 IT (Fig. 2F). Mechanistically, it is possible that the increased physical separation between these regions and the other repressive regions in the chromosome helped increase the transcriptional activities of these regions.

## Cell-type-dependent variations in A/B compartmentalization and their relationship with transcriptional activity

We also observed notable differences in A/B compartment arrangements across different cell types, in particular, between neurons and non-neuronal cells. Specifically, we observed stronger plaid patterns in the median pairwise distance matrices of chromosomes in neurons (fig. S8A) and performed further analyses to study these features.

**Fig. 3.**
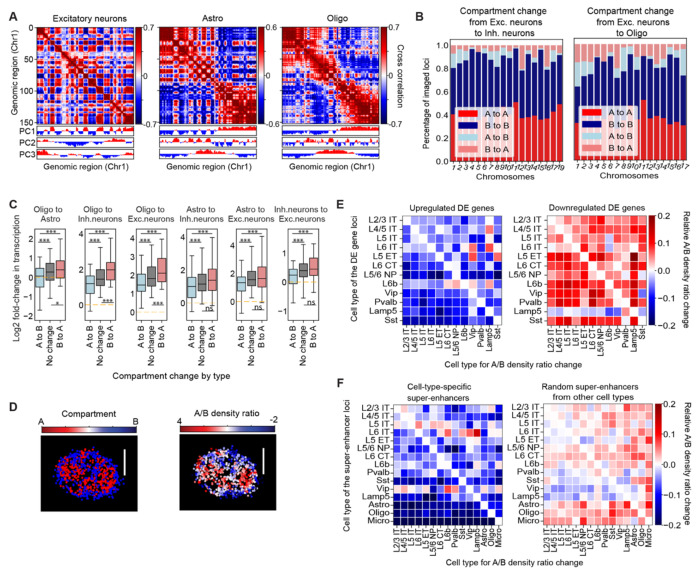
Cell-type-dependent variations in A/B compartment organization and transcriptional activity. (**A**) Cross-correlation matrices of Chr1 (3.7Mb – 98.8Mb) in several major cell types. Each element in the matrix represents the Pearson correlation coefficient between the indicated locus pair in terms of their normalized proximity frequency with all other loci from Chr1 (see Materials and Methods). The three rows below the matrices indicate the principal component (PC) values (positive values: red; negative values: blue). (**B**) Bar plots for the fraction of the A/B compartment conservations (e.g., A to A) and changes (e.g., A to B) from excitatory (Exc.) neurons to inhibitory (Inh.) neurons (left), or from excitatory neurons to oligodendrocytes and OPCs (right). Oligo here represents oligodendrocytes and OPCs together. (**C**) Boxplots for the distribution of the log2-fold change in transcription of individual genomic loci grouped by the A/B compartment conservations or changes from one cell type to another. The center line, box, and whisker represent the median, 25^th^-75^th^ percentile, and 5^th^-95^th^ percentile respectively. One-way ANOVA with Tukey post-hoc multiple comparison test was used for statistical analysis. ns: p>0.05; *: p<=0.05; ***: p<=0.001. (**D**) A single-cell image of compartment-A and B loci and their local A/B density ratio. Left: all decoded chromosome loci within a 2-μm z-plane in the nucleus, with compartment-A loci shown in red and compartment-B loci shown in blue. Right: same loci as in the left panel but colored by the local A/B density ratio. The local A/B density ratio is defined as the ratio of the local densities of trans-chromosomal A and B loci (i.e., A and B loci from other chromosomes, see Materials and Methods). Scale bar: 5 μm. (**E**) Changes in local A/B density ratio for cell-type-specific differentially expressed (DE) gene loci between the cell types where the DE genes were identified and other cell types. For each cell type, an imaged locus is considered a DE locus if there is a DE gene within 100kb of this locus. DE genes were identified for one cell type against all the other cell types using snRNA-seq data (52). Left: each pixel in the heatmap represents the median values of the fractional changes in the A/B density ratio of all upregulated DE gene loci for each cell type (x-axis) over the reference cell type (y-axis) where the DE genes were identified. Right: similar to the left panel, but for downregulated DE gene loci. (**F**) Similar to (E), but for changes in local A/B density ratio for cell-type-specific super-enhancer loci (left) or a random set of other super-enhancer loci selected regardless of cell-type identity (right). Cell-type-specific super-enhancers were selected by cell-type-specific long stretch snATAC-seq signals (52) (see Materials and Methods).

We applied standard Principal Component (PC) analysis to determine A/B compartments (16) in all major cell types. For each cell type, we calculated the normalized median pairwise distance matrix, computed the correlation matrix from this normalized distance matrix, performed PC analysis, and then selected the PC that showed the highest correlation with chromatin accessibility measured by snATAC-seq (52) to determine A/B compartments in that cell type (Materials and Methods). Interestingly, we found that the largest PC (PC1) did not always correspond to the A/B compartments in all cell types (Fig. 3A). For example, in excitatory neurons, the correlation matrix of Chr1 exhibited a pronounced plaid pattern, indicative of A/B compartment arrangements (Fig. 3A). Among all PCs, PC1 of the correlation matrix showed the highest correlation with chromatin accessibility (Fig. S10). In contrast, in oligodendrocytes and oligodendrocyte progenitor cells, PC1 primarily corresponded to the megadomain structures described above, whereas PC2 showed the highest correlation with chromatin accessibility (Fig. 3A and fig. S10). Astrocytes displayed an intermediate behavior, where megadomain structures and A/B compartments were similarly prominent (Fig. 3A) and both PC1 and PC2 showed similar correlation coefficients with chromatin accessibility (fig. S10). These results indicate that the megadomain structures and A/B compartments, which represent different types of higher-order chromosomal structures, differ in strengths between different cell types, apparently counteracting each other. Given that neurons had higher overall transcriptional activities than non-neuronal cells (Fig. 1, E and F), the observation that neurons showed more prominent A/B-compartment chromatin segregation suggests a possibility that a stronger degree of A/B compartmentalization may cause higher transcriptional activity or vice versa, for example through the formation of transcriptional condensates (6, 8).

In addition to the different degree of overall A/B-compartment chromatin segregation in different cell types, we observed switching of genomic loci from one compartment to another when the cell type changed (Fig. 3B). Between excitatory and inhibitory neurons, ~10-20% of genomic loci changed their compartment identities (A-to-B or B-to-A) (Fig. 3B, left). Likewise, between non-neuronal and neuronal cells, 10-30% of genomic loci changed their compartment identities (Fig. 3B, right). Interestingly, when changing from non-neuronal cells to neurons, or from inhibitory to excitatory neurons, transcriptional activity of genomic loci all tended to increase in general, regardless of whether the compartment identity of genomic loci switched from A to B, or from B to A, or did not switch (Fig. 3C). However, the genomic loci that switched from B to A compartment in general showed a greater increase in transcriptional activity than those that did not switch or switched from A to B compartments (Fig. 3C). These results suggest that A/B compartment rearrangement substantially influenced, but was not the sole determinant of, transcriptional activity change between cell types.

Next, we further explored the relationship between the local A/B chromatin environment and transcriptional activity of specific genes. To this end, we computed, for each imaged genomic locus, the local spatial densities of A and B chromatin loci near that locus and then determined the ratio of A-chromatin density over B-chromatin density as an indicator for the local A/B chromatin environment for that genomic locus in each cell (Fig. 3D; Materials and Methods) (24). This in turn allowed us to calculate, for each chromatin locus, the median of local A/B chromatin density ratio among all cells in any given cell type, which provided a quantitative proxy of the local A/B chromatin environment for each locus in each cell type.

We then investigated how this local A/B chromatin environment influenced differential expression of genes across different cell types. To this end, we first identified top differentially expressed (DE) genes (~200 upregulated genes and ~200 downregulated genes) in each cell type versus all other cell types using snRNA-seq data (52) (Materials and Methods). Among these top DE genes, we selected ones that were within 100kb of each of our imaged genomic loci, and then calculated the change in the median local A/B density ratio of this locus between the cell type where the DE gene was identified versus other cell types. Interestingly, we found that genes that were upregulated in a cell type in general tended to have higher local A/B density ratios in that cell type as compared to other cell types (Fig. 3E, left; fig. S11A; fig. S11B, left). Likewise, genes that were downregulated tended to have lower local A/B density ratios in that cell type (Fig. 3E, right; fig. S11A; fig. S11B, right).

We also examined the relationship between the local A/B chromatin environment and the activity of super-enhancers, which are known to contribute to cell-type-specific activation of gene expression (55, 56). For each imaged super-enhancer locus (which were all selected in a cell-type-specific manner), we examined the change in the local A/B density ratios between the cell type where the super-enhancer was identified and the other cell types. Similar to the trend observed for the DE genes, the super-enhancers also tended to have higher local A/B density ratios in their respective cell type as compared to other cell types (Fig. 3F, left; fig. S11C, left). In contrast, this trend was not observed in a randomization control where the super-enhancers were randomly selected regardless of cell-type identity (Fig. 3F, right; fig. S11C, right). Taken together, these results suggest that the local A/B-compartment chromatin environment plays a role in regulating cell-type-specific gene expression, not only for genes themselves, but also for gene regulatory elements.

## Cell-type-dependent variations in nuclear radial positioning of active and inactive chromatin

**Fig. 4.**
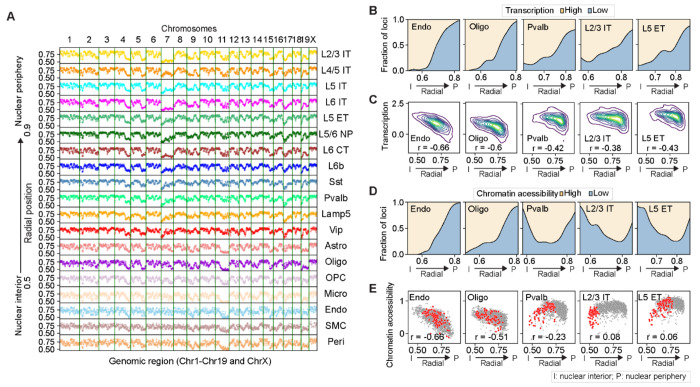
Cell-type-dependent variations in nuclear radial positioning of active and inactive chromatin. (**A**) Cell-type-median nuclear radial positions of all imaged loci (across Chr1-19 and ChrX, each column representing one chromosome) for individual cell types (rows). For each locus, nuclear radial position was calculated as the normalized distance of the locus to the centroid of the nucleus, with the maximum locus-to-centroid distance along the same direction normalized to 1. (**B**) The fractions of genomic loci with high and low transcriptional activity as a function of the normalized nuclear radial position for several cell types. Genomic loci were binned by their normalized radial positions. Within each bin, the fractions of high- and low-expression loci (top 25^th^ and bottom 25^th^ percentile, respectively, calculated from snRNA-seq data (52)) over the total counts of high- and low-expression loci were plotted. (**C**) 2D density plots of the cell-type medians of transcriptional activity of individual genomic loci versus the cell-type medians of normalized nuclear radial positions of the same loci. The density plot shows the kernel density estimation of the distribution of the data points. (**D**) Similar to (B) but for chromatin accessibility instead of transcriptional activity. The fractions of highly and lowly accessible loci were calculated from snATAC-seq data (52). (**E**) Scatterplots for the cell-type medians of chromatin accessibility of individual genomic loci versus normalized nuclear radial positions of the same loci. Gray dots represent loci from all chromosomes, and red dots represent loci from Chr7. The Spearman correlation coefficient r is indicated in (C) and (E).

We calculated the normalized radial positions of our imaged loci (normalized by the radius of the nucleus in the same direction, Materials and Methods) and observed distinct radial positioning for different genomic loci in different cell types (Fig. 4A). In general, neurons and non-neuronal cells showed lower similarity in nuclear radial positioning profiles of genomic loci than the similarities observed among subtypes of neurons or among non-neuronal cell types (fig. S12).

Interestingly, we observed different degrees of correlation between transcriptional activity of genomic loci and their nuclear radial positions for different cell types. In non-neuronal cells, transcriptional activity of genomic loci was strongly correlated with their nuclear radial positions, with loci positioned closer to the nuclear periphery exhibiting lower transcriptional activity (Fig. 4, B and C). However, this correlation was weaker in neurons, accompanied by increased transcriptional activity for genomic loci near the nuclear periphery (Fig. 4, B and C). This difference between neurons and non-neuronal cells was even more pronounced for the correlation between chromatin accessibility and nuclear radial positioning (Fig. 4, D and E). Moreover, the local A/B-chromatin environment of the imaged loci (as measured by their local A/B density ratio) were also less correlated with their nuclear radial positions in neurons as compared to non-neuronal cells (fig. S13). Interestingly, compared to excitatory neurons, inhibitory neurons showed consistently low levels of correlation between the nuclear radial positioning of genomic loci and their local A/B density ratios (fig. S13). Together, our results showed that active and inactive chromatin tended to be better segregated along the radial axis in non-neuronal cells than in neurons.

Interestingly, we also observed different degrees of correlation between transcriptional activity and nuclear radial positioning, and between chromatin accessibility and nuclear radial positioning, for different chromosomes (fig. S14, A to C). Notably, for some chromosomes, such as Chr7, the correlation between nuclear radial positioning and chromatin accessibility even showed opposite trends for non-neuronal cells and neurons (Fig. 4E; fig. S14, B and D). To understand the potential biochemical basis underlying these divergent behaviors between neurons and non-neuronal cells, we examined the histone modifications associated with our imaged loci, by examining single-cell joint histone modification and gene expression profiles obtained from the mouse cortex using Paired-Tag assays (63). We found that H3K9me3, a histone modification that marks heterochromatin (64), also showed opposite trends for radial-position dependence between neurons and non-neuronal cells (fig. S14, E and F). Likewise, such opposite trends were also observed for H3K27ac (fig. S14F), a histone modification that marks active chromatin (64). Therefore, compared to non-neuronal cells, neurons have more active chromatin in the nuclear periphery and more inactive chromatin near the nuclear interior, in part due to the inverted radial organization of a subset of chromosomes.

Along with the increased transcriptional activity of genes near the nuclear periphery in neurons, we observed that the majority of the transcriptionally active genomic loci in the nuclear periphery of neurons contained actively expressed long genes (>300kb) (fig. S15A), by combining published snRNA-seq data (52) with our spatial data from DNA-MERFISH (Materials and Methods). Many of these long genes are associated with gene ontology (GO) terms that are related to synapse functions (fig. S15, B and C). In the nuclear interior, although long genes were not preferentially activated over short genes in neurons, there were still more transcriptionally active loci containing actively expressed long genes in neurons as compared to non-neuronal cells (fig. S15A).

## Radial-position-dependent transcriptional regulation by MeCP2

**Fig. 5.**
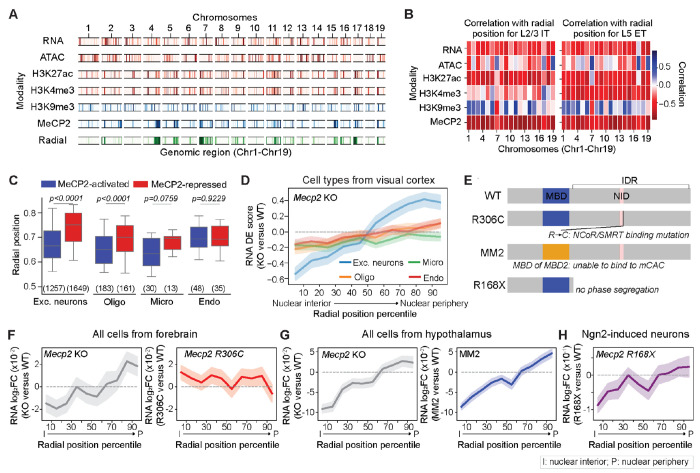
Nuclear-radial-position dependent transcriptional regulation by MeCP2. (**A**) Transcriptional activity (RNA), chromatin accessibility (ATAC), histone modifications (H3K27ac, H3K4me3, and H3K9me3), MeCP2-binding, and nuclear radial positioning for individual genomic loci on Chr1-19. For radial positioning, loci near the nuclear interior (35th percentile) are highlighted in color. For the other plots, loci enriched for the indicated signals (top 30th percentile) are highlighted in color. Transcriptional activity and chromatin accessibility were calculated from Ref. (52); H3K27ac, H3K4me3, and H3K9me3 levels were calculated from Ref. (63); MeCP2-binding level was calculated from Ref. (65). (**B**) Correlation of transcriptional activity, chromatin accessibility, histone modification levels, and MeCP2-binding level with nuclear radial positioning for L2/3 IT (left) and L5 ET neurons (right). The Spearman correlation coefficient between the radial positions and the indicated quantities of all loci on the indicated chromosome are shown. (**C**) Boxplots for nuclear radial positions of genes grouped by their transcriptional activity changes upon *Mecp2* deletion for various major cell types. Only genes that were significantly differentially expressed between male *Mecp2 +/y* (wild-type, WT) and *Mecp2 −/y* (knock-out, KO) mice (adjusted *p*<0.05), determined using data from Ref.(71), are plotted. Numbers on the bottom indicate the number of significant DE genes. *P* values indicate the statistical significance calculated by Mann Whitney U test, corrected by the Benjamini-Hochberg procedure. (**D**) Transcriptional changes upon *Mecp2* deletion versus the normalized nuclear radial positioning of the genomic loci for various cell types. For each gene, the radial position is approximated by that of the closest imaged locus and all mapped genes are grouped into 10 equal bins by their normalized nuclear radial positions. The transcriptional level change (RNA DE score) is defined as the t-statistic score for differential expression when comparing the expression-level distributions of *Mecp2* KO and WT cells. The mean (line) and 95% confidence interval (shade) for the DE scores in each bin are plotted. (**E**) Schematics of Mecp2 mutations/transgenes analyzed in (F to H). MeCP2 contains a methyl-CpG (mCG) binding domain (MBD, blue) and a NCoR/SMRT-interacting domain (NID, pink). *Mecp2* R306C mutation in the NID (68, 73) abolishes its interaction with the NCoR/SMRT complex, which is a transcriptional repressor. MM2 transgene (74) replaces the MBD of MeCP2 with the mCG-binding domain of the MBD2 protein, which abolishes MeCP2’s ability to bind to methylated CAC (mCAC) but not mCG. *Mecp2* R168X mutation (41) truncates the intrinsically disordered region (IDR) in MeCP2, which abolishes its liquid-liquid phase segregation ability. (**F**) Similar to (D), but for the transcriptional changes (log2 fold change (FC) of RNA counts) upon *Mecp2* KO (left) or *Mecp2* R306C mutation (right) measured in the mouse forebrain (73). (**G**) Similar to (F), but for the transcriptional changes upon *Mecp2* KO (left) or upon introduction of the *Mecp2* MM2 transgene (right) in mouse hypothalamus (74). (**H**) Similar to (F), but for the transcriptional changes upon *Mecp2* R168X mutation in Ngn2-induced cultured neurons (41).

Our observations showed that neurons and non-neuronal cells have different radial organization of active and inactive chromatin in the nucleus. To further understand the neuron-specific radial organization of chromatin in the nucleus, we compared the patterns of several active and repressive chromatin marks and chromatin-binding proteins across all imaged loci (Fig. 5, A and B). Interestingly, among all factors that we examined, the binding of the Methyl-CpG binding protein 2 (MeCP2) (65) consistently showed a high correlation with the nuclear radial positioning of chromatin in neurons, with a stronger tendency of MeCP2 binding to chromatin loci closer to the nuclear center (Fig. 5, A and B).

MeCP2 plays important roles in regulating gene transcription (66–71) and mutations of the *MECP2* gene cause the Rett syndrome, a progressive neurodevelopmental disorder (39) . Considering that MeCP2 is highly expressed in neurons (72), we next asked whether the correlation between MeCP2 binding and nuclear radial positioning of chromatin is related to the transcriptional regulation by MeCP2 in neurons. As a first step, we analyzed published scRNA-seq data from wild-type (WT, *Mecp2 +/y)* and *Mecp2* knock-out (KO, *Mecp2 −/y)* male mice (71). We focused on four major cell types from this dataset: excitatory neurons, oligodendrocytes, microglia, and endothelial cells because the number of inhibitory neurons and astrocytes measured in this dataset is relatively low. We found that genes downregulated upon *Mecp2* deletion (genes activated by MeCP2) preferentially localized closer to the nuclear center as compared to the genes upregulated upon *Mecp2* deletion (genes repressed by MeCP2) (Fig. 5C). Moreover, we systematically quantified the nuclear-radial-position dependence of transcription regulation by MeCP2 by calculating the mean differential-expression (DE) scores (t-statistics score) upon *Mecp2* deletion for all genes in these four cell types, using our imaging results to infer nuclear radial positions of the genes. Remarkably, we found that excitatory neurons showed a strong nuclear-radial-position dependence of transcriptional regulation by MeCP2: transcription of genes at the nuclear periphery were repressed by MeCP2 whereas genes near the nuclear interior were transcriptionally activated by MeCP2 (Fig. 5D). Interestingly, compared to excitatory neurons, the degree of transcriptional up- or down-regulation by MeCP2 was much smaller in non-neuronal cells (Fig. 5D).

To gain more insights into the molecular mechanisms underlying the radial-position-dependent effect of MeCP2, we further examined the radial-position-dependent effects of various *Mecp2* mutations by analyzing bulk RNA sequencing data (Fig. 5E). As an approximation, we mapped the genes in published bulk RNA sequencing data of *Mecp2* knockout and three *Mecp2* mutations (R306C, MM2 and R168X) (41, 73, 74) to the nuclear radial positions of the closest imaged genomic loci that we imaged. We observed a radial-position-dependent effect of transcriptional activity change upon *Mecp2* deletion in both forebrain and hypothalamus (Fig. 5, F and G, left), the general trend of which was similar to that observed for neurons in the scRNA-seq data (Fig. 5D). Next, we examined the effect of the R306C mutation in *Mecp2*, which disrupts MeCP2 binding to the transcriptional co-repressor NCoR/SMRT-complex (68, 73) (Fig. 5F, right). In contrast to *Mecp2* deletion, the R306C mutation caused a more moderate and largely radial-position-independent increase in transcriptional activity (Fig. 5F, right), indicating that the transcriptional upregulation by wild-type MeCP2 in the nuclear interior, as reflected by the decrease in transcriptional activity upon *Mecp2* deletion, does not require interaction with the NCoR/SMRT-complex. Likewise, the increase in transcriptional activity by the R306C mutation in the nuclear periphery was weaker than that by *Mecp2* deletion, suggesting that transcriptional repression by wild-type MeCP2 in the nuclear periphery is not fully accounted for by MeCP2’s ability to recruit the NCoR/SMRT co-repressor.

MeCP2 can bind to both methylated CG and methylated CA, with stronger affinity to mCAC than other forms of mCAH trinucleotides. To understanding which binding mode of MeCP2 is important for its radial-position-dependent transcriptional regulation effect, we analyzed bulk RNA sequencing data on the knock-in mice *Mecp2-MBD2* (MM2), which specifically abolishes MeCP2-binding to mCAC but not mCG by replacing the methylated DNA-binding domain of Mecp2 with that of the MBD2 protein (74) (Fig. 5E). Our analysis results showed that the MM2 transgene recapitulated the radial-position-dependent effect of *Mecp2* deletion on transcription (Fig. 5G, right).

MeCP2 is also a key component of the heterochromatin phase-separated condensates (41, 42). Our analysis of the RNA sequencing data on mice expressing the *Mecp2* truncation R168X (41), which lacks the intrinsically disordered region of MeCP2 required for liquid-liquid phase separation (Fig. 5E), showed that this mutation had a similar radial-position-dependent effect on transcription as seen in for *Mecp2* deletion (Fig. 5H).

Taken together, our analysis results suggest that MeCP2 regulates gene expression in a radial-position-dependent manner through its binding to mCAC and likely through the formation of phase-separated condensates. These molecular mechanisms promote transcriptional activation of genes in the nuclear interior and repress gene expression at the nuclear periphery. In addition, it has been shown previously that the ability of MeCP2 to recruit the repressive NCoR/SMRT-complex also contributes to MeCP2’s role in transcriptional repression (68, 73), and our data suggest that this latter effect occurs in a manner that is largely independent of the nuclear radial position. It is interesting to note that the R306C mutation confers milder symptomatic features in human patients than the R168X mutation or *MECP2* large DNA deletions (75). Hence, the radial-positioning-dependent transcriptional regulation by MeCP2 may be particularly important for the clinical severity in Rett syndrome.

## Effects of MeCP2 on nuclear radial positioning of chromatin and active/inactive chromatin segregation

**Fig. 6.**
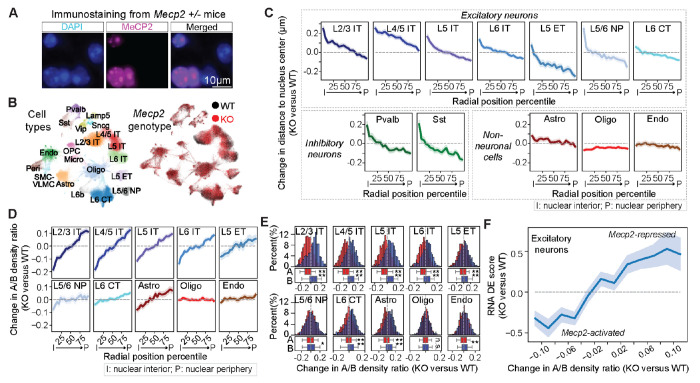
MeCP2-mediated A/B-compartment segregation regulates local chromatin environment in a radial-position and cell-type-dependent manner. (**A**) Representative images of DAPI (cyan) and MeCP2 (magenta) staining of a small region in the MOp in *Mecp2* +/− female mice. (**B**) UMAP representation of cell types (left) and genotypes (right) in the MOp of *Mecp2* +/− female mice. (**C**) Nuclear radial position changes upon *Mecp2* deletion as a function of the normalized radial position of the genomic loci in various cell types. All imaged loci were grouped into 20 equal bins based on their cell-type median normalized radial positions in the WT cells. (**D**) Similar to (C), but for the changes in local A/B density ratio upon *Mecp2* deletion. (**E**) Histograms of local A/B density ratio changes upon *Mecp2* deletion for compartment-A (red) and compartment-B (blue) loci in various cell types. The corresponding boxplots for the distributions are shown at the bottom. (**F**) Correlation between transcriptional changes and local A/B density ratio changes upon *Mecp2* deletion in excitatory neurons. Genes are grouped based on their local A/B density ratio changes indicated in the x-axis. For each gene, the differential expression (DE) score for transcription change between KO and WT cells was calculated as in Fig. 5D, using scRNA-seq data (71). For the line plots (C, D, F), the line and shaded area represent the mean and the 95% confidence interval, respectively. For boxplots in (E), the center line, box, and whiskers represent the median, 25^th^-75^th^ percentile, and 5^th^-95^th^ percentile, respectively. Student’s t-tests with Bonferroni corrections were used for statistical significance evaluation in (E). *: p<=0.05; ***: p<=0.001; ****: p<=0.0001; n.s.: p>0.05.

Given our observations of the correlation between nuclear radial positioning of genomic loci and their local A/B chromatin environment and transcriptional activity (Fig. 4 and fig. S13), and the radial-position-dependent transcriptional regulation by MeCP2 (Fig. 5, C to H), we wondered whether MeCP2 influences the nuclear radial positioning of the chromatin loci and their local A/B-compartment chromatin environment. To address these questions, we applied integrated RNA- and DNA-MERFISH imaging to measure chromatin organization in the MOp of *Mecp2* +/− heterozygous female mice. Since the *Mecp2* gene is on the X chromosome (39, 76) and one of two X chromosome homologs in female mice is randomly inactivated during development (77), we anticipate that roughly half of the cells in *Mecp2* +/− mice would express *Mecp2* from the WT allele and the remainder cells would not express *Mecp2* due to the KO allele. Through immunostaining of MeCP2 conducted in conjunction with MERFISH measurements on the same samples, we identified WT cells and *Mecp2* KO cells across different cell types (Fig. 6, A and B; fig. S16A). *Mecp2* deletion did not significantly change the cell type composition in the MOp (fig. S16B), nor did it change the size of the nucleus substantially, with only a subset of cell types exhibiting statistically significant but subtle changes in the nuclear volume (fig. S17).

Next, we systematically characterized the chromatin organization changes upon *Mecp2* deletion in different cell types. We focused our nuclear-radial-positioning analysis on several abundant cell types (fig. S18A). Notably, we observed a tendency for centrally-located chromatin loci to move towards the nuclear periphery and peripherally-located chromatin loci to move towards the nuclear center upon *Mecp2* deletion in most excitatory neurons and *Pvalb* and *Sst* inhibitory neurons (Fig. 6C; fig. S18B). Such movement was not observed in oligodendrocytes and endothelial cells and only to a small extent in astrocytes (Fig. 6C; fig. S18B).

Next, we examined the effect of *Mecp2* deletion on the local A/B-compartment chromatin environment of the imaged genomic loci. The cross-correlation matrices used to identify A/B compartments were highly similar between WT and *Mecp2* KO cells (fig. S19A), and the degree of similarity was particularly high for excitatory neurons, astrocytes, oligodendrocytes, and endothelial cells, but slightly lower for inhibitory neurons and microglia (fig. S19B). However, due to the low number of cells sampled for inhibitory neurons and microglia, we could not conclude whether these differences reflect real A/B compartment changes or noise. Therefore, we focused on the relatively abundant excitatory neurons, astrocytes, oligodendrocytes, and endothelial cells. Because of the high similarity between compartment boundaries observed between WT and *Mecp2* KO cells in these cell types, we used our A/B compartment assignment from the WT MOp datasets for further analysis.

Interestingly, similar to its effect on nuclear radial positioning, *Mecp2* deletion affected the local A/B chromatin environment of the imaged genomic loci most strongly in IT neurons, to a less extent in other excitatory neurons and astrocytes, but did not have any effect in oligodendrocytes and endothelial cells (Fig. 6D). In IT neurons, compartment-A loci showed a stronger tendency to move from the nucleus center outward to nuclear periphery compared to compartment-B loci, and this difference was reduced in other excitatory neurons and astrocytes, and further reduced or not observed in oligodendrocytes and endothelial cells (fig. S20). Consistent with these observations, the local A/B density ratio in excitatory neurons decreased for loci near the nuclear center and increased for loci at the nuclear periphery (Fig. 6D). This change was most pronounced in IT neurons, weaker in other excitatory neurons and astrocytes, and largely absent in oligodendrocytes and endothelial cells (Fig. 6D).

In line with the above observations, in excitatory neurons, compartment-A loci generally showed a decrease in local A/B density ratio and compartment-B loci generally showed an increase in local A/B density ratio (Fig. 6E), suggesting that A loci were relocated to a more heterochromatic environment and B loci were relocated to a more active chromatin environment upon *Mecp2* deletion. This trend was again the strongest in IT neurons, weaker in other excitatory neurons and astrocytes, but largely absent in oligodendrocytes and endothelial cells (Fig. 6E). Thus, *Mecp2* deletion led to an overall decrease in the degree of A/B-compartment chromatin segregation in excitatory neurons.

Finally, we observed a strong correlation between the changes in local A/B density ratio and transcriptional activity induced by *Mecp2* KO (Fig. 6F). Upon *Mecp2* deletion, the loci that showed an increase in local A/B density ratio also showed an increase in RNA expression level. Conversely, the loci that showed a decrease in local A/B density ratio showed a decrease in RNA expression level. Therefore, our data suggest that MeCP2 regulates the chromatin organization in neurons, in particular the nuclear radial positioning and A/B compartment segregation of chromatin, and through these effects, MeCP2 regulates transcription in excitatory neurons. Upon *Mecp2* deletion, the degree of A/B-compartment chromatin segregation was weakened, the nuclear interior became a less active environment, and the nuclear periphery became less repressive environment. This provides a uniform, mechanistic explanation for the apparently divergent effect of MeCP2 deletion on transcriptional activity, namely the decrease in transcription for genomic loci located in the nuclear interior and increase in transcription for genomic loci located in the nuclear periphery.

## Discussion

In this work, we developed an integrated RNA and DNA genome-scale imaging method to identify cell types and determine cell-type-specific 3D-genome organization in complex tissues. We applied this approach to study the primary motor cortex in the mouse brain and revealed a wide spectrum of nuclear-architecture differences among different brain cell types, including differences in the cell-nucleus sizes, chromosomal-territory sizes, higher-order chromatin structures, and nuclear radial positioning of chromatin. We further observed strong correlations of these cell-type-dependent variations in nuclear architecture and 3D-genome organization with transcriptional regulation, in terms of both the overall transcriptional activity of the cells and the differential expression of specific genes between cell types. Furthermore, our studies provided mechanistic insights into how MeCP2, a methylated-DNA binding protein, regulates the 3D-genome organization and transcription in neurons.

First, we observed that different cell types in the brain exhibit substantially different nuclear sizes and that the overall transcriptional activity of cells vary dramatically in a manner that is strongly correlated with the nuclear sizes (Fig. 1, D to F). In particular, both the nuclear size and the transcriptional activity increased from non-neuronal cells to inhibitory neurons, and then to excitatory neurons, and finer changes were also observed among subclasses of cells within these major cell classes. Our results suggest that the size of the cell nucleus could be a hallmark of the cell types and states. In disease contexts such as cancer, alterations in the nucleus size have been frequently reported and used as diagnostic markers (78). It is therefore possible that these disease-related changes in nuclear sizes also influence gene expression in a manner that is relevant to disease phenotypes.

Interestingly, as the nuclear size increased from non-neuronal cells to neurons, chromatin was not simply decompacted in a uniform manner. Instead, we observed a divergent trend in cis-chromosomal proximity for chromatin loci separated by different genomic intervals (Fig. 2, A to C), consistent with sequencing-based studies of the mouse and human brains reporting that non-neuronal cells and neurons exhibit distinct preferences for cis-chromosomal contacts at different genomic distances (28, 29, 31). In particular, despite their larger nucleus and chromosomal territory sizes as compared to non-neuronal cells, neurons exhibited a higher frequency of contact or spatial proximity for loci separated by short genomic intervals (<2Mb). This phenomenon may be attributed to the active transcriptional regulatory mechanisms in neurons such as the increased frequency in the formation of gene-body domain and enhancer-promoter loops (26, 29, 79). Interestingly, during the cell cycle as cells progress from G1 to G2/S phases, the transcriptional upregulation is also accompanied by an increase in the contact frequency of chromatin loci separated by short genomic intervals, whereas the contact frequency of long-genomic-interval locus pairs decreases (80). Thus, the increased interactions between loci separated by short-genomic intervals and decreased interactions between loci separated by long-genomic intervals maybe be a general signature associated with global transcription upregulation and nuclear volume increase in a variety of conditions, such as cell-cycle progress and cell-type differentiation (31, 80).

Notably, in non-neuronal cells, which exhibited relatively low overall transcriptional activity, chromosomes tended to form large-sized domain structures (Fig. 2, A and D), which resembled the megadomains previously observed in the transcriptionally inactive X chromosomes (20, 22, 60–62). Megadomain formation in inactive X chromosomes is mediated by the prevalent repressive epigenetic signatures, which compact heterochromatin into large domains separated by insulating elements (61, 62). Whether a similar mechanism causes the formation of megadomain structures in autosomes in non-neuronal cells remains an open question. By contrast, megadomain structures were diminished in neurons, which instead exhibited more prominent A/B compartments (Fig. 3A). The formation of megadomain structures and A/B compartments thus appear to counteract each other. Such an opposing effect has also been reported during X chromosome inactivation (22, 61). We hypothesize that the switching between megadomain and A/B-compartment structures are causally linked to transcriptional regulation, and that this switching is a general mechanism applicable to not only X chromosomes but also autosomes.

We also observed a correlation between the local A/B-compartment chromatin environment and transcriptional activity changes of specific genes between cell types (Fig. 3, E and F). Previous studies suggest that local A/B compartmentalization is associated with differential gene expression during brain development and aging (28, 30). Here, we showed that genes differentially expressed between different cell types were in general more strongly expressed in cell types where the local A/B chromatin density ratio of the genomic locus was higher. We observed the same trend for enhancer activity -- super-enhancers also tended to be more active in cell types where their local A/B density ratio was higher. The local enrichment for active chromatin could result in a higher local concentration of transcription machinery and activation factors, thereby enhancing the activity of these genes and enhancer elements. Conversely, it is also possible that active promoters and enhancers tend to colocalize with each other, for example, through the formation of transcriptional condensates (6, 8), thereby causing a higher local concentration of active chromatin.

Another major difference in 3D-genome organization that we observed between different cell types was the nuclear radial positioning of active and inactive chromatin. While non-neuronal cells exhibited a strong correlation between nuclear radial positioning of chromatin loci and their accessibility and transcriptional activity, with active and inactive chromatin respectively residing in the nuclear interior and periphery, neurons exhibited a more intermixed organization of active and inactive chromatin along the radial axis of the nucleus (Fig. 4, B to E). The widespread upregulation of gene expression in neurons may contribute to the enrichment of active chromatin in the nuclear periphery. The majority of active genomic loci near the nuclear periphery of neurons contain long genes (fig. S15A), which are likely more capable of concentrating transcriptional machinery (81), potentially overriding the repressive mechanisms in the nuclear periphery. In line with our observations, it has been shown recently that lamina-associated domains are less repressive in neurons than in cultured embryonic stem cells (82). Upregulation of transcription near the nuclear periphery may also weaken the tethering of inactive chromatin to the nuclear envelope (83, 84), causing its relocation to the nuclear interior.

Finally, we observed that MeCP2 regulates both chromatin organization and transcriptional activity in a nuclear-radial-position and cell-type dependent manner (Fig. 5 and Fig. 6). Notably, MeCP2 repressed transcription of genes residing near the nuclear periphery and activated transcription of genes near the nuclear center, and these effects were strongest in excitatory neurons and nearly absent in endothelial cells and oligodendrocytes (Fig. 5). Structurally, MeCP2 promoted local A/B-compartment chromatin segregation: upon *Mecp2* deletion, the originally active environment of nuclear interior underwent a decrease in local A/B chromatin density ratio, becoming less active, whereas the originally more repressive environment of nuclear periphery underwent an increase in the local A/B chromatin density ratio, becoming less repressive (Fig. 6). This role of MeCP2 in chromatin organization provides a natural explanation for the radial-position-dependent transcription regulation by MeCP2 and resolved a longstanding puzzle of why MeCP2 activates transcription of some gene and represses transcription of other genes (43, 44, 67, 69–71).

MeCP2 may enhance A/B-compartment chromatin segregation by promoting heterochromatin condensation. It has been shown previously that MeCP2 can cause liquid-liquid phase separation (41, 42) and that MeCP2-positive heterochromatin condensates do not coalesce with Brd4 and Med1 condensates that are enriched for active chromatin (41). This exclusion effect between MeCP2 condensates and transcription condensates could provide a mechanism to explain why loss of MeCP2 leads to increased intermixing between active chromatin and inactive chromatin, as we observed, and thereby causes transcription mis-regulation. Indeed, we observed that truncation of the intrinsically disordered domain in MeCP2, which abolishes its ability to form condensates (41), recapitulated the radial-position-dependent effect on transcription by *Mecp2* deletion (Fig. 5H). Since neurons exhibited a more intermixed organization of active and inactive chromatin along the radial axis (Fig. 4), the expression of MeCP2 may be particularly important for preventing unwanted crosstalk between active and inactive chromatin and thereby ensuring proper expression of genes in neurons. This cell-type-dependent effect of MeCP2 may thus reflect a functional adaptation to cell-type-specific 3D-genome organization.

In summary, our integrated RNA- and DNA-MERFISH studies provided rich insights into the connections between cell-type-specific 3D-genome organization and transcription regulation both in normal brain function and in disease conditions caused by *Mecp2* mutations. The ability of this technology to determine cell-type-specific 3D-genome organization in native tissues can be broadly applied to advancing our understanding of gene-expression regulation in health and in disease. We anticipate that future studies combining 3D-genome and transcriptome imaging with epigenome imaging (85) and protein imaging could further enhance our ability to understand the inter-relationship between epigenetic properties of chromatin, gene-regulation factors, chromatin structures, and transcription regulation. Moreover, combination of this approach with live-cell imaging, for example by CRISPR-based live-cell chromatin imaging (86) followed by fixed-cell genome-scale imaging of the same sample, and with spatial manipulations of genomic loci by CRISPR-based methods (87), could further advance our ability to study the causal relationship between 3D-genome organization and transcription regulation with high spatiotemporal resolution.

## Supplementary Material

Supplement 1

Supplement 2

Supplement 3

4

## Data Availability

Oligonucleotide probe sequences used for this study can be found in Tables S1, S2, and S3, and they can be purchased from commercial sources. Data reported in this paper are available at 4DN Data Portal (https://data.4dnucleome.org/; accession numbers: 4DNESPE924IP and 4DNESMTNNB3N). Analysis code used for MERFISH probe library design, MERFISH image decoding, and data quantification is available at: https://github.com/ZhuangLab/Chromatin_Analysis_2023.
